# Could palliative sedation be seen as unnamed euthanasia?: a survey among healthcare professionals in oncology

**DOI:** 10.1186/s12904-023-01219-z

**Published:** 2023-07-19

**Authors:** E. Lucchi, M. Milder, A. Dardenne, C. Bouleuc

**Affiliations:** 1 Department of Supportive and Palliative Care, Institut Curie, Saint-Cloud, France; 2 Department of Clinical Research, Institut Curie, Paris, France; 3 Department of Supportive and Palliative Care, Institut Curie, Paris, France

**Keywords:** Palliative care, End-of-life, Euthanasia, Sedation, Legislation

## Abstract

**Background:**

In 2016 a French law created a new right for end-of-life patients: deep and continuous sedation maintained until death, with discontinuation of all treatments sustaining life such as artificial nutrition and hydration. It was totally unprecedented that nutrition and hydration were explicitly defined in France as sustaining life treatments, and remains a specificity of this law. End- of-life practices raise ethical and practical issues, especially in Europe actually. We aimed to know how oncology professionals deal with the law, their opinion and experience and their perception.

**Methods:**

Online mono-centric survey with closed-ended and open-ended questions in a Cancer Comprehensive Centre was elaborated. It was built during workshops of the ethics committee of the Institute, whose president is an oncologist with a doctoral degree in medical ethics. 58 oncologists and 121 nurses—all professionals of oncological departments -, received it, three times, as mail, with an information letter.

**Results:**

63/ 179 professionals answered the questionnaire (35%). Conducting end-of-life discussions and advanced care planning were reported by 46/63 professionals. In the last three months, 18 doctors and 7 nurses faced a request for a deep and continuous sedation maintained until death, in response to physical or existential refractory suffering. Artificial nutrition and even more hydration were not uniformly considered as treatment. Evaluation of the prognosis, crucial to decide a deep and continuous sedation maintained until death, appears to be very difficult and various, between hours and few weeks. Half of respondents were concerned that this practice could lead to or hide euthanasia practices, whereas for the other half, this new law formalised practices necessary for the quality of palliative care at the end-of-life.

**Conclusion:**

Most respondents support the implementation of deep and continuous sedation maintained until death in routine end-of-life care. Nevertheless, difficulty to stop hydration, confusion with euthanasia practices, ethical debates it provokes and the risk of misunderstanding within teams and with families are significant. This is certainly shared by other teams. This could lead to a multi-centric survey and if confirmed might be reported to the legislator.

**Supplementary Information:**

The online version contains supplementary material available at 10.1186/s12904-023-01219-z.

## Key messages

i) What is already known about the topic? Studies have explored the ethics of choices, such as withholding or withdrawing treatments, moral positions to euthanasia, sedation practices or evaluation of end-of-life prognosis, but none have explored, in real life, health care professionals’ opinion and experience of all these aspects combined, in palliative cancer care.
ii) What this paper adds: This study highlights the discrepancy between some elements of the law and opinions of carers, along with the risk of conflict within teams and with families. It also highlights the risk of drifting towards euthanasia.
iii) Implications for practice, theory or policy: this study could lead to a multi-centric national study and could, if confirmed, add the voice of professionals to the ethical debate on end-of-life practices in France, which could be reported to the legislator. It could also lead colleagues of abroad to analyse practices in regard of their national laws on end-of-life.

**Supplementary Information:**

The online version contains supplementary material available at 10.1186/s12904-023-01219-z.

## Background

Concerns about the end of life generate impassioned debates. The time is long past when someone could write about euthanasia “deciding where to draw the line has sufficed to exclude the whole subject of discussion” [1]. As Campbell [2] traces it as a logical path, the scope of the right to die under advance directive legislation was primarily limited for patients to refuse unreasonable obstinacy or life extending medical treatments. Then came request assistance from a physician. Then it lead to patient decisions to request direct physician administration of a lethal agent when the underlying indication is not terminal illness but non-relievable pain and suffering. This latter ethical terrain, said the author, which historically has been designated by the concept “euthanasia,” has been re-conceptualized to *medically-assisted dying* (MAD) to avoid historical associations attached to “euthanasia.” Palliative sedation is one the possibilities to reduce intolerable suffering at the end of life, by using medications to reduce consciousness of patients with limited life expectancy. It may refer to a continuum of practice, ranging from the use of low doses of sedatives to deep sedation [3].The European Association for Palliative Care (EAPC) developed a 10-point framework for high-quality palliative sedation, completed by guidance from international experts [3, 4]. Pain, dyspnoea, delirium are the most common symptoms requiring sedation, while sedation for existential distress is more controversial [5, 6]. Palliative care clinicians express divergences about the extent to which sedation can hasten death, or the distinction between sedation and euthanasia, especially when the sedating medication is increased disproportionally, or when sedation is used for patients with a life expectancy of more than a few days [7–9]. Many nurses also worry about elements of decision, depth of sedation, its potential for shortening life, and the loss of social interaction [10, 11]. Palliative sedation to relieve existential refractory symptoms can be a source of suffering for them and highlights the need for pluri-professional team consultation [12, 13].

A few years ago, the French government initiated a debate about the end of life, with a view to revise a 2005 act. Thinkers from various backgrounds supposed to represent the thought of the French population were associated. The President of the National Assembly opened the draft of this forthcoming law to public contributions. Written on the Internet, they were listed, analysed and forwarded to the proposers [14]. In 2016 the so called Claeys-Leonetti Law (N° 2016–87) appeared. Its decree was set up in July 2016. It created new rights for patients. One of the most important was the possibility to ask for deep and continuous sedation until death. We add “maintained” until death because it introduces the notion of evaluating the level and efficacy of this sedation, so we will write deep and continuous sedation maintained until death (DCSMD). Three conditions are required: refractory suffering, incurable disease, and short-term prognosis. This DCSMD should be implemented with simultaneous discontinuation of all other treatments except painkillers. Artificial nutrition and hydration are considered as treatments. The law also insists on patients' right to refuse unreasonable obstinacy, on physicians’ obligation to do everything possible to relieve the physical suffering, even if it could shorten life, and on advance directives. They appear to be the best way for patients to express their end-of-life care with opposability to doctors. The major articles of the law are set out in annex 1, and the main components summarised in Table 1.

**Table 1:** Mains components of the French law « Claeys-Leonetti » about end-of-life care

Patients’rights	Terms and conditions
Person of trust	• Explain patient’s wishes concerning end-of-life care when the patient is not able to speak any more • Applicable with a written consent signed by both the patient and the person of trust • Can be modified or cancelled by the patient at any time • Physicians have to explain if they do not follow his/her advice
Advanced directives	• Two models, with or without serious illness • Written patient’s wishes concerning end-of-life care • Applicable with a written document signed by the patient • Can be modified or cancelled by the patient at any time • No validity period • Opposability to doctors • Physicians have to explain if they do not follow the advanced directives
New patient’s right : to ask for deep and continuous sedation until death	Applicable if the patient presents 3 conditions : 1. A refractory suffering 2. An incurable disease 3. A short term prognosis The decision has to be made during a multiprofessionnal meeting with 2 physicians at least. All treatments must be stopped excepted pain killers. Nutrition and hydration are considered as treatments

In February 2019 our ethics committee offered a training session on the clinical implementation of this law. It initially appeared that it was difficult to define refractory suffering. Secondarily, the definition of “short-term prognosis” was considered to be very ambiguous. Finally, the similarities and difference between DCSMD and euthanasia were hardly discussed. Accordingly we decided to conduct a trans-sectional survey of all oncologists and nurses in the hospital, aiming to explore doctors’ and nurses’ knowledge, opinions and experience of this act. We thought this survey could also contribute to the vibrant debate on euthanasia, which involves ethical, moral, religious and political questions.

## Methods

This is a mono-centric opinion survey, conducted in a French Cancer Comprehensive Centre. The ethics committee for the medical care of patients and relatives, whose president is an oncologist with a doctoral degree in medical ethics, built a questionnaire during workshops. The questionnaire used in our study was entirely developed for this study and is readable as an additional file. Its translation is the responsibility of the authors. The first part contained questions concerning the healthcare professionals’ experience of the application of the law. The second part explored their opinion concerning the terms of the law, the meaning of short- term prognosis, and the potential link, or not, with euthanasia. Some questions differed slightly between doctors and nurses, due to an adaptation to practices. Some questions of the survey partially mirror a previously published patient survey [15]. All definitions were written on the questionnaire, especially those regarding euthanasia, in conformity with those of the French Senate. The questionnaire was built using RedCap, a secure web application for building and managing online surveys and databases.

We sent an e-mail invitation to fill out the questionnaire once a week for three weeks, during the first quarter of 2020. The population consisted of oncologists and radiotherapists (n = 58), nurses and their managers, working day or night in oncological hospitalisation wards and day hospital (n = 121).Participants had three months to answer the questionnaire. All responses were anonymised. Statistical analysis was performed from January 2021 to June 2021. All analyses were performed using the statistical programming language R, version 3.6.0 (R Project for Statistical Computing). The variables were described by the numbers of each response method and by their percentage calculated for all the responses provided (exclusion of non-responses). A proportion test or Fisher's exact test were used to compare the distributions between groups of respondents. The significance level was set at five percent**.**

## Results

Completion rate was 35% (respectively 22/58 practitioners and 34/121 nurses, *p* = 0.23). The median age of doctors was 37 years (30-62), and that of nurses 29 (23-56). Median years practicing after speciality graduation was 10 (2-33) for doctors and 5 for nurses (1-35), with a *p* value 0,015.Only 5 nurses had been practicing for 10 years or more. No practitioners benefited from supervision or practice analysis, whereas 8 nurses did (0 versus 23.5%, *p* = 0.017). Supervision is a professional feedback on practices, focused on the assessment, improvement and development of the supervisee's knowledge, skills and behaviours in the practice of the profession. It is carried out with the support of a psychologist or a psychiatrist. In France it is recommended by a French ministerial directive (099–250,308) for palliative care teams. A larger proportion of doctors than nurses were males (8/22 versus 1/34, *p* = 0.0015).With regard to religion or spirituality, the group was homogeneous (proportion of believers: 38.2% of nurses, 50% of practitioners, *p* = 0.2, atheists and agnostics also well-balanced, respectively defined as someone who denies existence of God and someone sceptical about metaphysics and religion).

The main results concerning oncology doctors’ and nurses ‘experiences are presented in Table 2. The person of trust was checked by 37 respondents under 56*, id est* 66%. Physicians and nurses conducted frequent discussions about end-of-life issues with patients, (20/22 and 26/34, *p* = 0.28). They could help patients to write advance directives, doctors more often than nurses (14/22 versus 8/34, *p* = 0,004). Most participants considered this as a hard task, requiring time to listen to and answer patients and call on the palliative team. Analysis of the open-ended questions reveals the clinical circumstances of such discussions, which differ from doctors to nurses. Doctors include in their discussion technical elements related to the disease: futility of a new regimen of chemotherapy, uncertainty of benefit-risk ratio with an overly high risk of severe side effects, short term vital risk. Nurses focused on their perception of patients' expectations, severe symptoms and emotional status. In regard to the timing of these discussions, doctors suggested also that it would be better not to initiate these discussions in an emergency context, because it has to be anticipated, gradual and repeated. Nurses ‘answers are spread over a wider range: some of them said: “sooner is better”, others highlighted the need to answer the patient’s questions in singular timelines: patients give the timing, and one nurse even said it is so important to write such advance directives, that it should be done “without having an illness”.

**Table 2:** Experience of physicians and nurses from oncology and palliative care team concerning the French law « Claeys-Leonetti » about end-of-life care

N total caregivers = 56	Physicians *n* = 22	Nurses *n* = 34	*p*-value
Checking patients’ person of trust in files systematically very often	**2**	**9**	**ns **
	**12**	**14**	**ns**
Informing patients about the law			
Systematically or very often	**5 **	**8 **	**ns **
Occasionally or rarely	**13 **	**8 **	**ns **
Never	**3 **	**8 **	**ns**
No response	**1**	**0**	**ns**
Conducting frequently with patients discussions about end of life care	**20 **	**26 **	**ns **
Helping patients to write advanced directives	**14 **	**8 **	**0,004 **
Asking for help of palliative team in this area of end of life care	**6 **	**12 **	**ns **

Nine doctors and 7 nurses faced, at least once in the last three months, a DCSMD request. Nurses seemed to have difficulties defining what constitutes refractory suffering (27 did not answer the question, while all physicians did). Participants thought that each patient defines for themselves what refractory suffering is. The context of these DCSMD was physical refractory suffering in two thirds of cases, followed by existential pain. What is the “correct time” to talk to a patient about this sedation was a hard question for all. All doctors except one organised a collegial meeting as required by law. According to physicians, the person who has the most weight in the decision-making process is either the team's opinion or their own, rarely the patient’s (6, 2 and 1/9 respectively). They stopped nutrition systematically (6/9) or frequently (3/9), but they did not easily stop hydration: only 1/9 did that systematically, while 5 did that frequently and 3 rarely or never. For nurses, the most important reason for stopping nutrition or hydration was the opinion of the doctor who conducted the collegial discussion, or the team’s, then the patient’s.

The main results concerning doctors’ and nurses ‘ opinions are presented in Table 3. Participants’ opinion was based on complete reading of the law only in 50% of cases. Doctors read the entire act at least one time less often than nurses (6/22 versus 22/34; *p* = 0.013). Participants were favourable to DCSMD (52/56, 93%), in the strict context described by the law. They globally considered that stopping a treatment, and non initiating it, are different concepts (47/56, 84%). Considering hydration and artificial nutrition as a treatment rather than care is controversial for doctors and nurses, and with differences between them (respectively 8/22 versus 23/34, *p* = 0.03, for hydration; 16/22 and 33/34, *p* = 0.05, for total nutrition, 10/22 versus 26/34, *p* = 0.024, for partial nutrition).

**Table 3:** Opinions of physicians and nurses from oncology and palliative care team concerning the French law « Claeys-Leonetti » about end-of-life care

	Physicians (*n* = 22)	**Nurses (*n* = 34) **	***P* value **
Reading the entire law	**6 **	**22 **	**0,013 **
Deep sedation :			
Favourable	**19 **	**33 **	**ns **
Hostile	**1 **	**1 **	
No response	**2**	**0**	
Refractory suffering criteria* (multiple choices available)			
Unbearable pain	**17 **	**31 **	**ns**
Patient defines it	**16 **	**17 **	**ns**
Mental suffering	**12 **	**23 **	**ns**
Severe alteration of the global status	**4 **	**1 **	**ns**
visible tumor lesions on the body	**0 **	**1 **	**ns**
Extensive tumor on the face	**1 **	**4 **	**ns **
Nutrition as a treatment in the setting of end-of-life care : Yes when			
Total artificial nutrition	**16 (80%) **	**33 (97%) **	**0,05 **
Partial artificial nutrition	**10 **	**26 **	**0,024 **
Parenteral nutrition	**15 **	**31 **	**0,03**
Enteral nutrition	**13 **	**25 **	**ns**
No Response	**2 **	**0 **	**ns **
Hydration as a treatment in the setting of end-of-life care :			
Yes	**8 **	**23 **	**0,03**
No	**12 **	**11 **	**ns**
No response	**2 **	**0 **	**ns**
Stopping a treatment or not initiating it :			
It is the same	**3 **	**4 **	**ns**
It is different	**18 **	**29 **	**ns**
No response	**1 **	**1 **	**ns **
Short term prognosis^b^			
About a month	**3 **	**6 **	**ns **
About 2 weeks	**3 **	**2 **	**ns**
About 1 week	**3 **	**1 **	**ns**
Agony phase	**2 **	**0 **	**ns **
Impossible to give an estimation	**10 **	**24 **	**0,04**
No response	**1 **	**1 **	**ns **
Considering direct euthanasia^c^ versus indirect euthanasia^a,c^			
It is the same	**2 **	**5 **	**ns**
It is different	**19 **	**28 **	**ns**
No response	**1 **	**1 **	**ns **
Considering direct^c^ euthanasia versus passive euthanasia^e^			
It is the same	**1 **	**2 **	**ns**
It is different	**20 **	**31 **	**ns**
No response	**1 **	**1 **	**ns **
Considering indirect euthanasia^d^ versus passive euthanasia^e^			
It is the same	**8 **	**4 **	**ns**
It is different	**13 (59%) **	**29 (85%) **	**0,042**
No response	**1**	**1 **	**ns **
Does the law « open a door » to indirect euthanasia ?			
Yes	**14 **	**16 **	**ns**
No	**7 **	**18 **	**ns**
No response	**1 **	**0 **	**ns **
Does the law « open a door » to passive euthanasia ?			
Yes	**12 **	**15 **	**ns**
No	**9 **	**19 **	**ns**
No response	**1 **	**0 **	**ns **

^a^refractory suffering means symptoms which remain intolerable despite the patient receiving best known and possible care. In the questionnaire we asked (translation by authors) What do you define as refractory suffering? We proposed in a multiple choice question :

Unbearable pain despite best available treatment/ Intense mental suffering / Tumor lesions of the face / One or more visible tumor lesions / deep alteration of the general status / It is the patient who defines it

^b^In the questionnaire we asked (translation by authors): In your opinion, what is the appropriate time period to talk about the "end of life" (of the "short term") in article 3 of the law when it is written "...when the decision of the patient, suffering from a serious and incurable disease, to stop a treatment, will affect his/ her short-term vital prognosis, and is likely to cause unbearable suffering". We proposed in a multiple choice question :

the agonic phase/ about a week / about two weeks / about 3 weeks to a month / I don't know how to estimate this time

On the questionnaire, before answering, persons had three definitions in conformity with those of the French Senate (translation by authors) :

^c^Direct or active euthanasia is defined as deliberate administration of lethal substances with the intention of causing death, at the request of the patient who wishes to die, or without his or her consent, on the decision of a relative or medical professionals.

^d^Indirect euthanasia is defined as use of painkillers whose secondary and unintended consequence is death.

^e^Passive euthanasia is defined as refusal or withdrawl life-sustaining treatments.

The “short-term vital prognosis”, appeared difficult to assess, even impossible for some doctors and nurses (respectively 10/22 versus 24/34, *p* = 0.04). When respondents attempted to specify the corresponding life expectancy of this term, it varied from few hours to one month. Doctors stated they did not use any palliative prognostic index. Direct and indirect or passive euthanasia were clearly different for the majority of oncology nurses and doctors. In contrast, determining whether indirect and passive euthanasia are distinct was not so easy, despite they read the definitions before answering. Approximately half of all caregivers estimated that DCSMD opened the door to euthanasia, indirect (30/56, 53%) or passive (27/56, 48%). Two opposite opinions co-exist: considering DCSMD as a way to shift toward MAD practices, and considering that this practice may improve end-of-life care avoiding euthanasia. The verbatim reflects these two opposite views: “Sedation is masked euthanasia”, “the law just legalised terminal sedation”.

## Discussion

To the best of our knowledge, our study is the first survey to assess the implementation of a law on end-of-life issues among health professionals in a cancer centre in France. There is no opposition to the law. Half of participants consider existential distress as a valid indication to ask for DCSMD. This last point is a positive finding, as preview studies showed that this kind of suffering is not often taken into account also it should be [16–18]. Our study shows participants are used to having discussions about end-of-life care and 50% have experimented DCSMD over the past three months. However, the timeline is difficult to assess: when should end-of-life discussions be initiated and how is a short-term prognosis estimated? Doctors insist on clinical features and nurses more on patients’ questions to define the “best moment”, if there is one; and this task is the responsibility of doctors, in order to respect the patient’s values and preferences. Studies have shown these discussions can reduce aggressive treatments, provide end-of-life care that is more concordant with the patient’s preference and increase early referrals to hospice [19]. Many participants reported that they had difficulties clearly defining the prognosis for patients with terminal illness. They have divergent appreciation of it, with a broad range from agony to one month. However, prognosis assessment for advanced cancer patients is notoriously difficult [20–23]. An extensive review of prognostic factor studies in oncology (from 17 oncology journals with an impact factor above 7) highlighted frequent over-interpretation and misreporting [24].

Contrary to the law, some respondents consider hydration and nutrition – especially when partial – to be a care, rather than a treatment, this difficulty to define these two supports was also shown by other authors [25–27]. The use of hydration is a topic for European guidelines. For example, it is continued in Norway if the patient was drinking more than 500 ml/day before sedation. Studies show that it is continued for 1/4 to 2/3 of patients. Evaluation of its continuation according to side effects should take place [28, 29]. Some participants in our study may then experience difficulties discontinuing hydration and/ or nutrition when DCSMD is initiated. To let patients “die of hunger and thirst” is also unbearable for loved ones who consider artificial nutrition and hydration as beneficial if a patient has not sufficient oral intakes [30], even at the end of life. To stop hydration and even nutrition may create a value conflict for a caregiver, which is known to be a risk factor for burn-out syndromes [27, 31, 32]. Furthermore, for more than 80% of participants, withdrawing and withholding treatments appeared to be different, which is consistent with previous studies [33]. The debate about the equivalence or non-equivalence between withholding and withdrawing treatments is complex, underlying formal ethics and practical considerations. This debate has its historical roots in analyses of the ethical relation between acts and omissions. For some authors [34], both are ethically equivalent, mostly because they are both decisions, and lead to the same results. They insist on what they consider as the real question, *id est* “whether or not treatment should be provided—not whether treatment has previously been started “. On the other hand, accepting in all situations the equivalent thesis could lead to medical ethics based on utilitarianism, which denies caregivers’ psychology and satisfaction [35]. Both professionals ‘positions might co-exist in in-depth discussions with patients and eventually relatives, when the prognosis remains uncertain, if the patient has clearly expressed their wishes, or even when faced with a scarcity of resources.

Despite long-standing attempts to define sedation at the end of life [36, 37], lots of different definitions remain in studies as Kremling A et al. [38] point it out, and that can lead to confusions. In line with this confusion, the major finding of our study is the divergence among participants concerning the link between DCSMD and medically assisted dying (MAD). In Europe, positions toward euthanasia range from a complete ban to conditionally authorised procedures, which are summarised in Table 4. Approximately half of our caregivers thought that the law opens a door to indirect euthanasia and/or to passive euthanasia. The Gordian knot is temporality. If DCSMD is only applicable to patients in agony or dying within a few days, the law cannot be considered as a new right, DCSMD is a normal palliative care, a “sedation at the end of life” and not a “terminal sedation” [39]. If DCSMD is applied to patients with a life expectancy of a few weeks or more, then it could be considered as a never mentioned but real passive euthanasia, as the withdrawal of nutrition and hydration will hasten the patient’s death [30]; or even been proposed when patients meet criteria for MAD [40]. In a Dutch survey, Overbeek et al. [41] suggested that the legal distinction between euthanasia and palliative sedation may not always be clear in clinical practice and may be linked to life expectancy. The use of terms such as "palliative or terminal sedation" or "euthanasia", "assisted suicide", "ending of life" was dependent on life expectancy. “Palliative sedation” was more often used when patients had a short (1–7 days) or very short (< 24 h) life expectancy. However, in an interview study in the French speaking part of Switzerland, a country where assisted suicide (AS) is decriminalised, DCSMD appears generally not as an alternative to a AS [42]. There is no other legislation on DCSMD in other European countries, except a 2022 circular in the Netherlands, that reviews the very strict conditions of application of this patients’ right, especially intention, process and results. The prognosis of patients must not exceed 2 weeks, the following death is considered natural and the sedation is a care, contrary to euthanasia which is not a patient ‘right and the death considered as non natural. But Janssens et al. [43] criticised this point of view, as soon the Royal Dutch Medical Association's wrote first guidelines on palliative sedation, which lead to a revision and the circular of 2022. Guidelines, of the European Institute of Ethics in 2019, also come back to this practice, insisting on the prognosis of patients, which must be evaluated in days. The difference is major with the French law where the *short term* prognosis is not defined. In Norway, guidelines (not a law) have also removed the prognosis estimate from 2 weeks to an indefinite period “at the end of life”.

**Table 4:** Various legislation in Europe

**Legalisation of active euthanasia**	**Decriminalisation of active euthanasia if medical assisted suicide not possible**	**Active euthanasia forbidden but possibility of assistance at the end of life to alleviate suffering, passive euthanasia accepted, and patients ‘right to refuse life sustaining treatments (avoid unreasonable obstinacy )**	**Active euthanasia and medical assisted suicide strictly forbidden by the law**	**Active euthanasia strictly forbidden by the law but possibility to request medical assisted suicide as an exception / or** **decriminalisation of it**	**Active euthanasia strictly forbidden by the law but medical assisted suicide not forbidden**	**Legalisation of medical assisted suicide**
**Netherlands,** **Belgium,** **Luxembourg,** **Spain**	**Portugal**	**Hungary, Czech** **Republic, Slovakia,** **Germany, France,** **Denmark, Finland, ** **Estonia, Sweden, ** **Croatia, ** **Switzerland, Greece,** **Slovenia, Norway,** **United Kingdom**	**Bulgaria,** **Poland, Cyprus,** **Malta, Latvia,** **Lithuania,** **Ireland, Czech** **Republic,** **Romania,** **Denmark,** **Hungary,** **Norway, United** **Kingdom **	**Italy/ Austria**	**Germany**	**Switzerland, **

https://www.touteleurope.eu/societe/l-euthanasie-en-europe/

Regarding sedation – we do not mean mild nor intermittent sedation but DCSMD -, the double effect, known since Saint Thomas of Aquinas, may alleviate consciousness and makes the ethical difference between DCSMD and MAD. It requires four points: the action itself must be good or morally neutral; the good effect must result from the act, not the bad effect; the bad effect must not be directly intended, but must be foreseen and tolerated, and the good effect must be stronger than the bad effect, or both must be equal (in the sense that it would be a greater evil to avoid it without producing the good effect). In case of sedation: if sedation is proportional, decreased consciousness or death is a non-intended bad effect, expressed intention remaining symptom relief as practices show in other European studies [25, 26, 44–46], and the bad effect is clearly not a means of achieving good effect. Intent and compliance with the medical deliberation procedure avoids shifting application of the law toward indirect euthanasia. Nevertheless, for Morita T et al. [47] this remains an ethical issue because real intention of practicians is never known, and because situations differ between imminently dying patients and not imminently dying ones. For the latter, death might not always be a bad outcome. For imminently dying patients, sedation is not justified by the principle of double effect if decreased consciousness is defined as bad effect, because its intention includes the bad effect and this decreased consciousness is a means of achieving the good effect. However, we can argue that sedation at the end of life, decided because of intolerable suffering, is normal care, as seen above. It alleviates suffering by decreasing consciousness, and the bad effect is death. Morita T et al. [47] counter-argues that some authorities do not support this theory due to the possibility of a co-existing intention to palliate symptoms *and* hasten death.

When we examine doctors’ intent, one European study reported that approximately half of decisions to withdraw or withhold treatments were intentionally done to hasten death [48]. Fontalis A et al. [49] mentioned also the possibility of a conflict of interest, for example, that “particularly in healthcare systems where doctors play the role of gatekeeper to healthcare, a doctor’s support for an assisted death might be negatively interpreted as a conflict of interest, with a desire to help relieve the social and economic burden of a patient’s illness upon society overriding the patient’s individual interests”.

We do not discuss patient’ demand, which is absolutely requested by our law, nor the question of patient’ autonomy, which is another subject.

We have examined proportional sedations. But there can also be a continuum between them and direct introduction of a maintenance dose without titration or slowly arbitrarily increased levels of sedation, which can appear as indirect euthanasia [17]. Because unresponsiveness induced by DCSMD does not necessary means awareness [50] the French law associate DCSMD with opioids: this combination of midazolam and opioids also raises the question of “slow “ or “covert” euthanasia, as E. Young et al. [51] pointed it out. Hahn MP [52] stress that the distinctions, in the use of the drugs in particular, are not recognised everywhere, when others even think that there is no difference [53], in a consequentialist position where same effect means same act, regardless of moral intention. In Belgium and the Netherlands, DCSMD could even have been performed as an alternative to euthanasia [3, 46, 54–56].

Maybe a fundamental frontier could be agnosticism [57] or a sense of transcendence: if only the value of life is at stake, including social life and capacity to communicate, then sedation is already death [58, 59]. If not, the distinction remains morally intact, as the ontological dignity of the human being must be respected until biological death [60–62]. Then it would be necessary to know patients’ spiritual positions, which is not routinely made, even if spiritual care is growing.

The line between palliative sedation and euthanasia remains thin. Citizens also have expressed their fear that these practices could be an open door or a disguised practice of euthanasia [63]. Moreover, our decision to stay within the law is frequently severely criticised by families, either because they would like us to perform active euthanasia or, at the contrary, because they blame us for practising a disguised form of euthanasia. Finally, this French right to DCSMD could be a disguised way to apply a more socially acceptable and unnamed form of indirect or passive euthanasia. Some authors [64] had raised the question before law’s decree by writing “Recognizing continuous deep sedation as a *sui generis* practice could remove the need to portray the practice either as symptom control or as a form of euthanasia”. It is an argument to evaluate its implementation before any further legislative changes are made.

## Conclusion

In pluralistic societies, with the increasing attention and demand for palliative care, new laws are being implemented to improve end-of-life care. However, there has been insufficient assessment of the real-life practices and opinions of healthcare workers, which directly impact the clinical outcomes of the new laws. This was the aim of this study. The results indicate that most respondents support the routine use of deep and continuous sedation maintained until death (DCSMD) in end-of-life care. The healthcare workers' comprehension of the law can reveal the adequacy of their education and training, while their opinions may reflect potential issues that require further clarification by the law, such as establishing a consensus on the definitions of refractory symptoms and short-term prognosis, which are not specified. Estimation of prognosis remains particularly difficult. However, some healthcare professionals expressed confusion regarding DCSMD and medical assisted dying, especially because this sedation is associated with opioids and withhold or withdrawal of nutrition and hydration. Withhold or withdrawal seem ethically different to the majority of practitioners. The sedation it-self, depending on how it is done, may be an open door to indirect euthanasia. The legislator might not have anticipated these conflicts. The results could also increase public awareness of the challenges and perspectives of healthcare professionals and raise important questions for further discussion. This study could serve as a guide for future debates and reviews of DCSMD. It leaves considerable scope to future research: conducting multicentric studies with larger sample sizes, involving other specialties such as palliative care teams, but also patients, and their caregivers. It also underlines the necessity to teach and share medical ethics.

## Limitations

The response rate was good for such a survey, but this remains a sensitive issue and may have introduced a bias. We are certain that one person could respond only once. Another limitation of this study was its mono-centricity and the size of samples, which can induce a bias like “spirit of the house”, which may not reflect the culture in other centres. Nevertheless, the difficulty to find funds for such studies limited its design for a multi-centric study. To go further, we are now beginning a qualitative study with patients at the end of their life, to understand how they decide their last will, and change eventually in the last weeks of life. A third step could include patients’ caregivers.

## Supplementary information

**Additional file 1.**

**Additional file 2.**

## Data Availability

Materials described in the manuscript, including all relevant raw data, and questionnaires, will be freely available to any scientist wishing to use them for non-commercial purposes, without breaching participant confidentiality, on request to the corresponding author.
