# Supplementary material for: Could palliative sedation be seen as unnamed euthanasia?: a survey among healthcare professionals in oncology
Source: BMC Palliat Care. 2023 Jul 19;22:97. doi: 10.1186/s12904-023-01219-z (PMC10354970; doi:10.1186/s12904-023-01219-z)
Supplement: Supplementary file 1 — Additional file 1. [file 12904_2023_1219_MOESM1_ESM.docx]

Annex 1 : major contributions in the 2016 law

- to affirm patients’ rights in the context of the end of life and the duties of doctors towards these patients (Article 1);

- to introduce a right to deep and continuous sedation maintained until death (DCSMD) at patient’s request, under specific conditions like refractory

suffering, and for people whose prognosis is vital in the short term. This sedation is done with simultaneous stop of all other treatments except painkillers

(Article 3);

- to insist on patients' right to refuse treatments, and to receive palliative care (Article 5);

- to clarify the conditions for stopping life sustaining treatments. This gives consideration to refuse an unreasonable obstinacy;

- to strengthen the scope of advance directives (Article 8); Advance directives have become the preferred expression of the wishes of patients who are

unable to express them, and are now binding on doctors. This change in the law concerning advance directives and their opposability to the doctor was the

most significant.

-to make explicit the status granted to testimony of the trusted third party (Article 9);

-to define the hierarchy of patient's wishes expressions. (Article10);

-to apply DCSMD during an incurable disease three conditions are necessary: patients request; “refractory suffering” and short-term life expectancy. (Article 4).
