# Supplementary material for: Could palliative sedation be seen as unnamed euthanasia?: a survey among healthcare professionals in oncology
Source: BMC Palliat Care. 2023 Jul 19;22:97. doi: 10.1186/s12904-023-01219-z (PMC10354970; doi:10.1186/s12904-023-01219-z)
Supplement: Supplementary file 2 — Additional file 2. [file 12904_2023_1219_MOESM2_ESM.pdf]

Gender, age, number of years in practice

Do you currently benefit from practice analysis or supervision?

Yes/ No

If yes : How long have you been receiving practice analysis or supervision*?

*Supervision is a professional feedback on practices, focused on the assessment, improvement and development of the supervisee's knowledge, skills and behaviours in the practice of the profession. It is carried out with the support of a psychologist or a psychiatrist. In France it is recommended by a French ministerial directive (099-250308) for palliative care teams.

With regard to your religious beliefs, do you consider yourself to be

(Definitions according to The Larousse, french dictionnary)
Atheist (who denies the existence of God)
Agnostic (who considers that the absolute is inaccessible to the human mind and advocates the refusal of any solution to metaphysical problems)
Believer (who has religious faith)
If yes : Would you say that you are a practising Christian?
Yes/ No
Other
Do not wish to answer

Can you tell us more?

You and the Claeys-Léonetti law

Since it has enacted in 2016, have you once read the entire text of the 2016 Claeys-Leonetti law?
Yes at least once /yes more than once /no never

In the patient's medical file, do you know who is the person of trust is?
Systematically /very often /often /occasionally /rarely/ never
As the referring oncologist, are you the one who informs your patients of their rights under the Claeys-Leonetti Act?
Systematically/ often or very often / occasionally / rarely/ never
As the referring oncologist, are you the one who initiates end-of-life discussions with your patients?
Yes/ No
How often do you think you have had end-of-life discussions with your patients in the last 3 months?
At what point in the illness do you think it would be appropriate to initiate these end-of-life discussions?
Do you initiate discussions with patients with a view to write advance directives?
Yes/ No
How often do you think you have had discussions about writing advance directives with your patients in the last 3 months?

When do you think it would be appropriate to start these discussions about writing advance directives?
If you have discussions about writing advance directives, or more generally about the end of life, do you refer the patient to a palliative care doctor or team?
Always when advance directives are to be written / Systematically/ Often/ Sometimes / Never
Would you say that you are in favour of the use of deep and continuous sedation causing an alteration of consciousness maintained until death, combined with analgesia and the discontinuation of all life-sustaining treatments in the situations described in article 3?
Yes/ No
In the last 3 months, have you referred to the Claeys-Leonetti law to make a decision on sedation at the end of life (use of a sedative to obtain a deep and continuous sedation maintained until death, combined with analgesia and the discontinuation of all life-sustaining treatments in the situations described in article 3)?
Yes/ No
How many times in the last 3 months do you think you have used the Claeys-Leonetti law in this context of deep and continuous sedation maintained until death in the event of refractory suffering in a patient at the end of life?
Did these decisions follow a formal team meeting?
Systematically /Often /Sometimes/ Never
In the last three months, in the context of such sedations, have you stopped artificial feeding?
Systematically/ Often/ Rarely /Never
In the context of such sedations, have you stopped nutrition on the advice of:
Of the patient as a priority/ of the patient's family as a priority/ of the care team as a priority/mine first
In the last three months, In the context of such sedations, have you stopped hydration?
Always/ Often/ Rarely/ Never
In the course of these sedations, have you stopped hydration on the advice of:
Of the patient as a priority / of the patient's family as a priority / of the care team as a priority / mine first

Nutrition

Do you consider artificial nutrition to be treatment or care?

	treatment	care
If it is entirely medicalised		
If it is partial		
If it is enteral		
If it is parenteral		

Do you consider hydration to be treatment or care?

Refractory suffering

What do you define as refractory suffering?
Un bearable pain despite best available treatment/ Intense mental suffering / Tumour lesions of the face / One or more visible tumour lesions / Deep alteration of the general status / It is the patient who defines it
In your opinion, what is the appropriate time limit for talking about the "end of life" (the "short term") in article 3 of the law? ("*...when the decision of a patient suffering from a serious and incurable condition to stop a treatment will affect his or her short-term vital prognosis, and is likely to cause unbearable suffering").*
The agonic phase/ about one week / about two weeks / about 3 weeks to a month / I don't know how to estimate this time.
What do you consider to be a decisive criteria for initiating deep and continuous sedation maintained until death?
Temporality /Refractory suffering perceived by the patient/ The patient's request / In my opinion, there is no hierarchy between the two.
In the last months of a patient's life, do you use prognostic scores to help you make medical decisions?
Systematically / Often / Sometimes / Never
If used: which one(s)?
Do you distinguish between "stopping" and "not starting" life-sustaining treatment?
Yes, they are two different things
Can you say why you think these are two different things?

No, they are two identical things
Can you say why you think they are the same thing?

Euthanasia

The Senate distinguishes between different types of euthanasia:
- Direct or active euthanasia = the deliberate administration of lethal substances with the intention of causing death, at the request of the patient who wishes to die, or without his or her consent, at the decision of a close relative or the medical profession.
- Passive euthanasia = refusal or withholding of life-sustaining treatment
- Indirect euthanasia = administration of painkillers, the secondary and unintended consequence of which is death.

What do You think about these different types of practices ?

	It is the same	It is different
Direct/ active *versus* passive		
Indirect *versus* passive		
Indirect *versus* direct/active		

Do you consider that the Claeys Leonetti law has opened the way for indirect euthanasia?
Yes /No
Could you tell us more?
Do you consider that the Claeys Leonetti law has opened the way for passive euthanasia?
Yes/ No

Can you tell us more?

Comments (if you wish)

For nurses and head nurses : Some questions were added or different

What position do you hold: nurse / head nurse

Questions beginning **by « as the referring oncologist began by « as a nurse… » »**

When we asked how artificial nutrition or hydration were stopped, « of the patient as a priority / of the patient's family as a priority / of the care team as a priority / mine first » was modified by

« of the patient as a priority / of the patient's family as a priority / of the care team as a priority / of **the referring oncologist as a priority / of the doctor leading the collegial discussion as a priority** ».

All other questions were the same.
